# Extreme Hyperleukocytosis in Meningococcal Bacteremia Mimicking Leukemia: A Case Report

**DOI:** 10.1155/crdi/4514407

**Published:** 2026-09-09

**Authors:** Camille Gorena, Borna Amir-Kabirian, Ju-Hsien Chao

**Affiliations:** ^1^ School of Medicine, University of Louisville, Louisville, Kentucky, USA, louisville.edu; ^2^ Department of Medical Oncology and Hematology, University of Louisville, Louisville, Kentucky, USA, louisville.edu; ^3^ Department of Blood Cancer, Cellular Therapeutics and Transplant, University of Louisville, Louisville, Kentucky, USA, louisville.edu

**Keywords:** hyperleukocytosis, Neisseria bacteremia, Neisseria meningitis

## Abstract

Hyperleukocytosis (> 100 × 10^9^/L) is typically associated with hematologic malignancies and is rarely reported in infection. A previously healthy 27‐year‐old man presented with acute gastrointestinal symptoms and was found to have meningococcal bacteremia complicated by thrombocytopenia, coagulopathy, and acute kidney injury. Despite appropriate antimicrobial therapy, his leukocyte count rose progressively, peaking at 173 × 10^9^/L on hospital day four. Bone marrow biopsy and cytogenetic studies excluded leukemia. With continued antibiotic therapy and supportive care, his leukocyte count, coagulopathy, and renal function gradually normalized over the following 10 days. This case highlights hyperleukocytosis as a rare manifestation of meningococcemia and emphasizes the importance of excluding malignancy while managing infection‐related complications.

## 1. Introduction

Hyperleukocytosis, defined as a white blood cell (WBC) count exceeding 100 × 10^9^/L, is most commonly associated with hematologic malignancies, particularly acute leukemias and chronic myeloproliferative neoplasms. This degree of leukocytosis represents a hematologic emergency due to the risk of leukostasis, disseminated intravascular coagulation (DIC), tumor lysis syndrome, and end‐organ ischemia [1].

In contrast, infection‐associated leukemoid reactions, typically defined as WBC counts > 40–50 × 10^9^/L with a marked left shift, are well described but rarely exceed 100 × 10^9^/L. Severe bacterial infections may provoke profound inflammatory responses. However, extreme hyperleukocytosis approaching levels seen in leukemia is exceptionally uncommon in sepsis and prompts urgent evaluation for an underlying hematologic malignancy [2].

Neisseria meningitidis is a well‐recognized cause of fulminant sepsis, frequently complicated by coagulopathy, thrombocytopenia, and multiorgan failure [3]. Although leukocytosis is common in meningococcemia, hyperleukocytosis exceeding 100 × 10^9^/L has not been reported to our knowledge. We describe a rare case of meningococcal bacteremia complicated by extreme hyperleukocytosis peaking at 173 × 10^9^/L, closely mimicking acute leukemia and necessitating extensive hematologic evaluation.

## 2. Case Presentation

**FIGURE 1:**
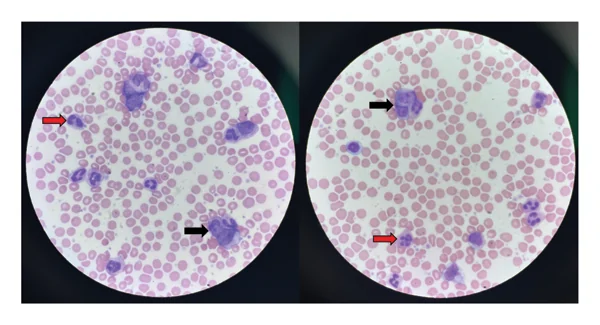
Peripheral smear at 50x magnitude depicting toxic neutrophils (red arrows) and myelocytes (black arrows).

A previously healthy 27‐year‐old man presented with acute‐onset nausea, vomiting, and diarrhea. He reported several weeks of night sweats but denied fever, headache, rash, or neurologic symptoms. On admission, laboratory evaluation revealed marked leukocytosis with a WBC count of 70 × 10^9^/L (reference range: 4.0–10.8 × 10^9^/L), with differential showing normal neutrophil predominance (60.4%, reference range 34%–75%) but concerning for an increased percentage of metamyelocytes (11.7%) and myelocytes (10.8%), both of which are normally absent. The remainder of the laboratory results were concerning for severe thrombocytopenia (32 × 10^9^/L; reference range 140–420 × 10^9^/L), prolonged prothrombin time (30 s; reference range 10.2–12.9 s) and partial thromboplastin time (70.4 s; reference range 26.6–35.9 s), and severe hypofibrinogenemia (54 mg/dL; reference range 230–450 mg/dL). A peripheral blood smear demonstrated granulocytosis with a left shift and thrombocytopenia (Figure 1).

Blood cultures grew Neisseria meningitidis, and broad‐spectrum intravenous antibiotics were initiated. The patient received supportive treatment in the intensive care unit for presumptive DIC. Despite appropriate antimicrobial therapy, he developed progressive thrombocytopenia, acute renal failure, and a rise in the leukocyte count, peaking at 173 × 10^9^/L on hospital day four. Given concern for acute leukemia, bone marrow biopsy was performed, and empiric all‐trans retinoic acid (ATRA) was initiated pending results.

**FIGURE 2:**
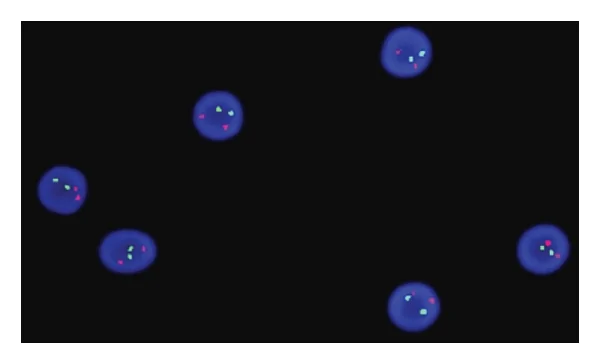
Fluorescence in situ hybridization (FISH) analysis performed using a specific set of probes for acute promyelocytic leukemia. Counts were within the normal reference range.

**FIGURE 3:**
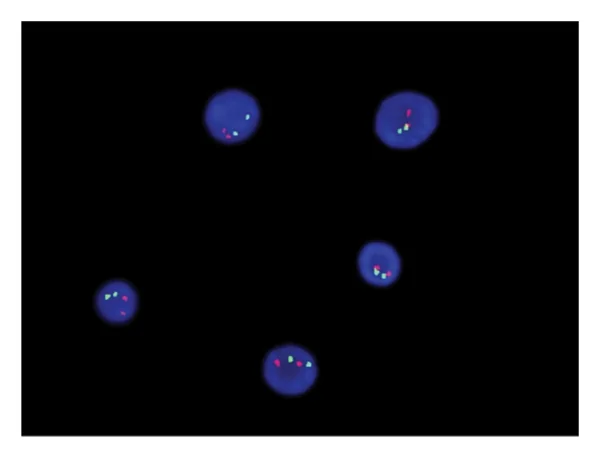
Fluorescence in situ hybridization (FISH) analysis performed using a specific set of probes for the BCR::ABL1 rearrangement associated with t (9;22) which was negative for the BCR::ABL1 rearrangement.

Bone marrow biopsy demonstrated normocellular marrow with multilineage hematopoiesis and left‐shifted myeloid maturation. There was no morphologic or immunophenotypic evidence of leukemia or lymphoma. Fluorescence in situ hybridization (FISH) was negative for acute promyelocytic leukemia (APL) and *breakpoint cluster region with Abelson gene* (BCR::ABL1) rearrangement (Figures 2 and 3).

Following continued supportive care and antimicrobial therapy, the leukocyte count gradually declined, reaching 14 × 10^9^/L by hospital day 14. Platelet counts recovered to 391 × 10^9^/L, renal function improved, and dialysis was discontinued. The patient was discharged in stable condition. At outpatient follow‐up, renal function had returned to baseline.

## 3. Discussion

This case illustrates an extreme leukemoid reaction secondary to severe meningococcal bacteremia, with leukocyte counts exceeding thresholds typically associated with hematologic malignancy. Leukemoid reactions are reactive, nonclonal proliferations of mature leukocytes and are most commonly triggered by infection, malignancy, or severe physiologic stress [2]. However, WBC counts surpassing 100 × 10^9^/L are exceedingly rare outside of leukemia and typically necessitate bone marrow evaluation [1, 4].

Several features of this case provided a diagnostic challenge. The continued rise in leukocyte count despite appropriate antimicrobial therapy raised concern for autonomous myeloid proliferation. Additionally, the presence of thrombocytopenia, coagulopathy, and hypofibrinogenemia suggested possible APL with DIC. In this context, empiric initiation of ATRA and expedited bone marrow biopsy were appropriate and consistent with current hematologic practice [4]. Definitive exclusion of leukemia allowed further management to focus on infection‐targeted treatment and supportive care.

Although leukocytosis is common in meningococcemia, most series describe leukopenia or modest leukocytosis in severe disease. This often reflects marrow exhaustion or consumption in fulminant sepsis [5]. Reports of extreme hyperleukocytosis in invasive meningococcal disease are exceedingly rare, highlighting the unusual nature of this presentation. The pathophysiology of such extreme leukocyte elevation in sepsis is incompletely understood but likely reflects exaggerated cytokine‐driven myelopoiesis, accelerated marrow release of immature granulocytes, impaired leukocyte margination, and delayed apoptosis in the setting of overwhelming inflammation [2, 6, 7]. Similar phenomena have been reported in severe infections, solid tumors, massive hemorrhage, and neonatal populations, though at substantially lower leukocyte counts [7].

The patient’s clinical course raises the possibility that marked leukocytosis contributed to end‐organ dysfunction, including acute kidney injury and coagulopathy. Hyperleukocytosis, regardless of etiology, has been associated with hyperviscosity, microvascular obstruction, endothelial injury, and secondary DIC. However, these complications are far better characterized in leukemia than in reactive states [1, 6]. In infection‐associated hyperleukocytosis, distinguishing cause from consequence remains challenging, and supportive management of sepsis and coagulopathy remains critical.

Management strategies for hyperleukocytosis in nonmalignant conditions are not well defined. While leukapheresis may rapidly reduce circulating leukocyte burden, evidence supporting its use in reactive leukemoid reactions is limited, and the procedure carries substantial risks in patients with thrombocytopenia or active coagulopathy [8]. Current expert opinion favors treatment of the underlying infection and meticulous supportive care, reserving cytoreductive interventions for exceptional cases with refractory organ dysfunction clearly attributable to leukostasis [1, 8].

## 4. Conclusion

This case expands the recognized hematologic manifestations of meningococcemia and demonstrates that extreme hyperleukocytosis approaching levels typically seen in leukemia can occur in severe bacterial infection. Awareness of this rare presentation is essential to guide appropriate diagnostic evaluation, avoid premature diagnostic anchoring, and ensure timely supportive management.

## Funding

The authors have no funding to disclose.

## Ethics Statement

This case report was exempt from institutional review board (IRB) review in accordance with institutional policy for single deidentified case reports.

## Consent

Verbal informed consent was obtained from the patient for publication of this case report and any accompanying images.

## Conflicts of Interest

The authors declare no conflicts of interest.

## Data Availability

Data sharing is not applicable to this article as no datasets were generated or analyzed during the current study.
